# Acculturation, Work-Related Stressors, and Respective Coping Strategies among Male Indonesian Migrant Workers in the Manufacturing Industry in Taiwan: A Post-COVID Investigation

**DOI:** 10.3390/ijerph191912600

**Published:** 2022-10-02

**Authors:** Wan-Chen Lee, Natasia Shanice Chanaka, Charng-Cheng Tsaur, Jiune-Jye Ho

**Affiliations:** 1 Institute of Environmental and Occupational Health Sciences, College of Public Health, National Taiwan University, No. 17, Xu-Zhou Road, Taipei 100, Taiwan; 2 Institute of Labor, Occupational Safety and Health, Ministry of Labor, Taipei 22143, Taiwan

**Keywords:** acculturation, Indonesian, migrant worker, manufacturing, COVID-19

## Abstract

Globalization has prompted cross-cultural migration in search of employment opportunities, and poor adaptation during acculturation is widely known to cause additional psychosocial stress. Hence, the objective of this study was to investigate migrant workers’ perceptions of acculturation, identify work-related stressors, and understand the respective coping strategies among male Indonesians in the manufacturing industry, particularly during the COVID-19 pandemic. Fifteen workers were recruited and interviewed on their acculturation experiences. We found that the workers were susceptible to forced adaptation to stressful conditions relating to work organization, economic distress, interaction with the manpower agencies, language barriers, and so on. During the pandemic, workers experienced overload, economic hardship, suspended home visits, isolation, discrimination, and fear of cluster infection in the crowded dormitory. We also found that workers were able to adopt coping strategies by capitalizing on resources at the individual, institutional, and governmental policy levels to actively solve problems, increase emotional support, and fortify self-appraisals. The identified coping strategies could inform policy development to assist with positive adaptation and promote the well-being of the migrant worker population.

## 1. Introduction

Globalization has prompted cross-cultural migration in search of employment opportunities and contributed to the 169 million global migrant worker population in 2019 [1]. Taiwan has an aging society and relies substantially on the workforce from Southeast Asia. In 2020, the population of migrant workers has increased by 0.67 times in the past decade and has reached more than 0.7 million [2]. While one in thirty-three of Taiwan’s population was a migrant worker, 60% of them were employed in the manufacturing industry due to limited educational background and widespread language barriers. These work opportunities often have 3D (dirty, dangerous, and demanding) characteristics, low skill requirements, and low wages, leading to higher psychosocial stress and increased risks of injury and illness among workers [3,4,5,6]. Specifically, the accident incidence rate was 2.87 per one thousand migrant workers, which consisted of 48% from Vietnam, 22% from Indonesia, 13% from Thailand, and 16% from the Philippines [2]. In addition to the type of employing industry, being male and Indonesian were found to be individual, significant factors in accident incidence rates, with the most common cause of being caught in machinery [7]. The high risk of injury could also be partly explained by psychological stress. Research in other countries has suggested significant associations between psychological distress and occupational accidents [8,9]. 

Acculturation arises when migrants of different cultural backgrounds are in contact with a new culture in the host country [10]. Poor adaptation during acculturation is widely known to cause additional psychological stress for migrants [11,12,13]. The acculturation process can be conceptualized based on Lazarus’ stress and coping theory and Berry’s acculturation-coping-adaptation model [14,15,16]. According to Lazarus, when encountering a stressor, an individual would undergo cognitive processes of primary appraisal to assess if the stressor poses a threat and secondary appraisal to evaluate one’s resources in response to the situation [15]. The latter directly links to the coping strategies categorized into problem-solving, emotion-regulating, and appraisal-oriented coping by Lazarus [14]. Adequate and effective utilization of these coping strategies helps achieve successful adaptation [14,17]. Berry classifies acculturation strategies individuals adopt into separation (maintaining the culture of origin and rejecting interaction with the host culture), marginalization (relinquishing all cultural identities), integration (maintaining the culture of origin and actively adopting the host culture), and assimilation (abandoning the culture of origin and adopting the host culture) [10,16]. Hajro et al. alluded to Berry’s acculturation strategies theory and developed a multilevel (individual, organizational, and societal) framework to link acculturation, coping, and integration success for international skilled migrants (ISMs) [18]. At the individual level, examples of the critical influences on the ISMs’ acculturation outcomes included motivation to migrate, demographic characteristics, and cross-cultural competencies. At the organizational level, integration policy, human resource management, and climate for inclusion played important roles in integration success. Finally, at the societal level, immigration policies, societal attitudes towards migrants and foreigners, and accreditation and recognition of foreign qualifications and work experience could influence how ISMs acculturate and cope. 

**Figure 1:**
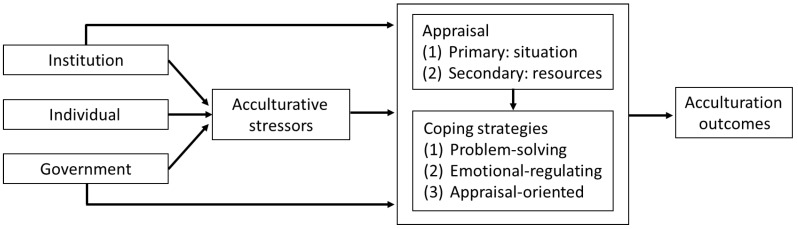
Conceptual framework of acculturation process (adapted from Lazarus, Berry, and Hajro et al.) [14,16,18].

Building on the aforementioned theoretical concepts and models [14,16,18], we developed a conceptual framework to explore the linkage of how characteristics and resources at the individual, institutional, and governmental policy levels could influence acculturation outcomes through various coping strategies among male Indonesian workers in the manufacturing industry (Figure 1). At the institutional level, we specifically focused on factors contributed by companies/factories in the manufacturing industry. 

The compounded effects of precarious work conditions and acculturative stress could render male Indonesian workers vulnerable to reduced physical and mental health. As a result, understanding their work-related and acculturative stressors and the respective coping strategies would help shape policies to provide the necessary resources to reduce psychosocial stress and increase safety. However, studies on stressors for industry workers in Taiwan mainly focused on language barriers, contributing factors to injuries and fatalities, and their OSH training status from dataset analyses [7,19,20]. No research has examined the coping strategies workers adopt to alleviate acculturative stress. Therefore, the objective of this study was to investigate workers’ perceptions of acculturation, identify work-related stressors, and understand the respective coping strategies among male Indonesians in the manufacturing industry, particularly during the COVID-19 pandemic. Findings from this study will provide information on effective coping strategies experienced by migrants to strengthen positive adaptation, which contributes to improving migrants’ health.

## 2. Materials and Methods

This study is part of a more extensive study aimed at understanding Indonesian migrants’ perceptions of work-related experiences and injury-related factors. Fifteen male Indonesian workers from the manufacturing industry were recruited between September and November 2021 from northern and southern Taiwan using the snowball and venue-based sampling methods, as well as through the network of staff in non-governmental organizations (NGOs). All participants worked in different companies and were interviewed outside their workplaces by a trained, multilingual Indonesian research assistant with a public health background. In addition to work-related experiences, they were specifically asked about acculturation processes, such as workplace relationships, stressors at work, how they arranged their time and activities outside work, and the relevant changes during the COVID-19 pandemic. All interviews were audio-recorded after the participants gave oral and written consent for the interview. The Indonesian research assistant transcribed the content of the audio recordings from Indonesian to English. The transcripts were subsequently coded using the same descriptive labels to categorize similar concepts for each question [21]. We further identified factors affecting workers’ psychosocial well-being and coping strategies based on the summarized concepts. The study design was approved by the Research Ethics Center of the National Taiwan University.

## 3. Results

The participants were employed in factories with tasks requiring intensive manual labor, such as shipbuilding, metal processing, welding, meat-cutting, and so on. They had worked in Taiwan for three to twelve years, but five had never visited home. Only one worker had returned to Indonesia more than three times. Workers have a wide range of experiences and perceptions regarding their work organization and conditions, physical environment, food choices, and acculturation outcomes. We presented their perceptions relating to psychosocial status in the following categories: workplace relationships, workplace violence, language barriers, sick leaves, activities outside work, other stressors, and the impacts of the COVID-19 pandemic. 

### 3.1. Relationships at Work

Workers experienced both positive and stressful relationships at work. Most participants indicated good relationships with their coworkers (regardless of nationality), supervisors, and employers. A worker pointed out that even though there was a language barrier, it did not cause problems:

*“I have a really good relationship with them. They are really kind to me and treat me as their children and friend. Sometimes there is a language barrier, but they would understand and explain to me patiently.”*

On the other hand, another worker explained his stress regarding workplace relationships:

*“…regarding the relationship with my boss or the foreman, it is not very harmonious. They do not really appreciate or respect our work. And when we need anything, they do not really show any effort to help us.”*

### 3.2. Workplace Violence

Three participants experienced physical violence in the workplace from abusive employers or supervisors. One worker reported the incident to the 1955 Hotline:

*“… I was physically abused. Thus, I recorded everything that happened and reported it to 1955.”*

The other two workers described their individual experiences:

*“I had a problem with my Taiwanese supervisor to the point where we physically fought. The incident was reviewed through the CCTV (closed-circuit television) security system, and he ended up being expelled.”*

*“In the previous factory, the boss was physically abusive and got mad easily. That is one of the reasons I shifted to my new job.”*

### 3.3. Language Barriers and Proficiency

Language barriers were prevalent in workers’ day-to-day lives and could cause inconvenience or stress at work. However, without additional Chinese lessons, an improvement in proficiency was difficult. A worker expressed his concern about this after ten years of employment in Taiwan:

*“I feel my Chinese ability has not improved because I could not talk with anyone.”*

In the face of such difficulties, another worker adopted a humorous viewpoint on poor Chinese ability and shared his perspective:

*“It is all good. Sometimes I become a comedian to the Taiwanese because of my Chinese ability.”*

On the other hand, good language proficiency contributed to better interpersonal relationships and helped solve problems, as pointed out by one worker:

*“I am already quite comfortable with the Taiwanese coworkers here because I could already understand some Chinese conversational words. I could also ask them for help if there are things I do not understand. My relationships with my boss and other coworkers are also good.”*

### 3.4. Sick Leaves

Enrollment in the National Health Insurance (NHI) program is mandatory for all migrant workers in Taiwan. When the workers felt unwell or tired, they could use their entitled days of sick leave. To access medical treatment, the workers would visit clinics or hospitals either by themselves or accompanied by Taiwanese coworkers, their supervisor, or the staff from the manpower agency. Most workers acknowledged the easy access to medical services:

*“Since we already have insurance, if the sickness is not that severe, we would just go to the hospital by ourselves.”*

The attitude towards clinical visits also varied among workers. One expressed resistance to seeking medical treatment:

*“If it is still bearable, we would just hold it. Our friends would help with our work so that it is not too heavy. We will not tell the boss because they would directly bring us to the clinic, while we Indonesians do not really like going to hospitals or clinics.”*

### 3.5. Other Stressors

We found that the manpower agencies were one of the main sources of stress for migrants in Taiwan. Due to the language barriers and unfamiliarity with the culture and regulations, workers relied on manpower agencies to help them with various problems, including changing jobs, interpreting or translating documents, renewing of their Alien Resident Certificate cards (an identity card to prove legal resident status), and medical emergencies. Under these circumstances, the agencies have reportedly asked for unreasonably large sums of fees, and often the workers felt they were “tricked” into paying more money. This created an additional financial burden, especially on those already in debt from borrowing money to pay the agency for employment. Two examples are demonstrated in the following excerpts:

*“My problems are usually related to the agency. They sometimes think that I do not have any knowledge, so they trick me by asking for a lot of money to extend my passport.”*

*“There was one time when I wanted to move to another factory, and the agency asked me to pay around NT$24,000. I felt it was a lot of money. Since I did not want to go back to Indonesia, I had to pay.“*

We also identified the scenarios where workers explicitly used the word “stress” (or “stressed”) in their narratives. The scenarios included being in a foreign country, hearing that their family was sick, experiencing heat and work overload, limited food options, disproportionality between salary and workload, and income insecurity during the COVID-19 pandemic. 

### 3.6. Activities Outside Work

Outside of work, the Indonesian workers turned to support groups and participated in personal, social, and religious activities. The most common responses from the interviews included gathering with friends, participating in activities through Indonesian social groups (e.g., organizations), and engaging in religious events (e.g., going to the mosque). Personal activities included fishing, cleaning or fixing the dormitory, messaging friends, calling family, and resting in the dormitory. A worker talked about his leisure time arrangement, which captured the general theme of activities shared by many Indonesian migrants off work.

*“I joined social activities such as fundraising, being active in organizations, and gathered with other Indonesian friends to share our daily lives.”*

A worker further addressed the importance of access to the public prayer rooms:

*“I am very grateful to the Taiwan government for providing us facilities to pray and do other religious practices before the pandemic since our factory does not have a praying facility.”*

Additionally, two workers had jobs in the restaurants.

### 3.7. Impacts of the COVID-19 Pandemic

Some workers experienced increased stress, where contributing factors were characterized by increased time pressure due to overload, economic hardship, suspended home visits due to border control, isolation under the company’s lockdown policy, discrimination, and fear of cluster infection in the crowded dormitory. Examples are demonstrated from the following excerpts:

*“We do not get any overtime work anymore, which affects our income. Meanwhile, we have to pay a lot of things aside from our living fees.”*

*“… it has been four years since the last time I met my wife and children.”*

*“I feel quite stressed because it is even harder to go out of the factory…”*

*“There are too many people in the dormitory, so there is a higher risk for us getting sick or COVID-19.”*

Although most responses referred to increased stress, we also observed examples with positive perceptions. One said:

*“I felt happier because there was less workload, but with the same amount of salary.”*

## 4. Discussion

### 4.1. Factors Influencing Acculturation

Migrant workers in Taiwan are susceptible to forced adaptation to the work environment and stressful conditions relating to work organization, economic distress, interaction with the manpower agencies, and acculturative processes. Many of these problems partly stemmed from structural issues at the policy level. For instance, migrant workers are constrained with regard to changing their jobs in Taiwan. To ensure workforce stability, the Employment Services Act stipulates that workers are not allowed to change jobs before their contracts end unless they can acquire the employer’s consent or present proof of extreme work conditions, such as the employer’s violation of the law. In the latter aspect, workers are requested to provide “evidence document of employment contract termination due to original employer’s plant closure, shut down or not paying work compensation according to employment contract” or “evidence document of other reasons whose responsibility cannot be ascribed to employed foreigners” to apply for change of jobs [22]. In other words, if the workers have problems at work which have not escalated to meet the requirements mentioned above, they are forced to adapt because they cannot afford to lose their jobs. The next challenge is that when the workers can meet these requirements, they often need to pay the manpower agencies additional fees to help with the administrative process. This further deepens the economic hardship among the workers, especially when they are still in debt to the agencies. Financial difficulties would subsequently prompt the workers to work overtime, reinforcing the pathway to an increased risk of injury [23]. To reduce such economic exploitation prior to employment, the Ministry of Labor established the Direct Hiring Service Center to assist employers in the manufacturing industry in hiring migrant workers on their own, bypassing the agencies. However, the additional administrative procedure might be perceived as inconvenient for employers in small and medium-sized enterprises.

Language proficiency plays an essential role in acculturation, and its barriers have been associated with workplace injuries for migrant workers [4,5,24]. Choi and Thomas found a positive correlation between acculturation attitude and fluency of the language used in the host country [25]. The ability to communicate in Chinese with coworkers and for daily needs affected the participants’ understanding of work-related issues and mental support. Improved language proficiency empowered workers to seek help from others when encountering difficulties.

### 4.2. Coping Strategies

This study found a wide range of acculturation outcomes (Table 1). Among the positive perceptions of adaptation, most workers reported good workplace relationships. In the previous study, social support from supervisors and coworkers was found to have protective effects on being injured in an occupational accident [26]. Regarding acculturative stress, Indonesian migrant workers shared similar experiences to those reported by the global migrant worker populations across different nationalities, host countries, and occupations [13,27,28,29,30,31]. Although a direct association between acculturative stress and occupational injury remains unclear, we believe it shares similar characteristics to the relationships between psychological distress and the risk of occupational accidents and injuries. 

We have summarized the different coping strategies workers adopted by the resources they could access at the individual, institutional, and governmental policy levels (Table 2). At the individual level, the various strategies were categorized into problem-solving, emotion-regulating, and appraisal-oriented coping [13,14,32]. Problem-solving is characterized by workers’ actions and attempts to change the situation, such as seeking help in interpreting, cooking by themselves to avoid pork-containing meals, taking a leave or utilizing healthcare services when feeling ill, and calling the 1955 Hotline to deal with physical abuse from the employer. Emotion-regulating coping mechanisms include workers’ engagement in religious and social activities for stress relief as well as contacting family or friends for support. In particular, religion and social networks play essential roles in this regard. Our findings are consistent with the previous study, where Fisher et al. found Muslims tended to seek social or family support in response to stressful situations [33]. We discovered that Indonesian migrants had strong community bonds, and many actively participated in social and religious activities. For instance, fundraising is a major activity for one social group, with donations from Indonesian workers across cities in Taiwan. These donations are used to support Indonesian migrants in the face of emergencies, to organize activities, and for charity purposes. For example, when migrants are involved in physical conflicts or break the law in Taiwan, the social group would lend migrants bail money for their release from detention in the police station. The social group also builds mosques and gives donations to help vulnerable populations, such as orphans, in Indonesia. Finally, in situations that workers had limited control over, they turned to appraisal-oriented coping strategies such as changing their attitudes (e.g., trivializing minor injury experiences, blaming oneself for carelessness, or believing everything depends on one’s “heart”) or resorting to humor (e.g., laughing at oneself) to “accept” the undesirable circumstances or risk at work. 

At the institutional level, specific company policies sometimes helped alleviate workers’ stress, including hiring interpreters, providing Indonesian food, having a functioning leave management system, and organizing activities. We found that manpower agencies play a double-sided role in acculturation. Chang et al. summarized the roles of manpower agencies as: to serve as a bridge of communication between workers and employers, assist in OSH training, and help workers adapt to life in Taiwan [34]. When the agencies fail to fulfill such roles, they become a significant source of stress for the workers. For example, agencies might be selective in providing interpreting services. During the interview, a participant expressed frustration dealing with manpower agencies and asked the researcher team for help with translating administrative and medical documents. 

The workers widely appreciated three governmental policies and incorporated the resources into the coping strategies at the individual level. The first one is mandatory enrollment in the NHI program, which is comparable to the policies in some European Union member countries [35,36]. Among the 480 migrant workers surveyed by Chu et al. in Taiwan, 93.5% reported having NHI coverage. In our study, the participants were willing to seek healthcare services when they felt ill. And when they did so, they mostly visited the medical facilities by themselves or with company. Similar findings were reported elsewhere [37,38].

Religion is a critical aspect of an Indonesian migrant’s life and can provide solace and support to cope with stress [39,40]. In addition to mosques in major cities, public prayer rooms can be accessed at the airport, major railway stations, tourist attractions, and freeway service areas [41]. There is also an increasing number of public prayer rooms in the harbor regions, set up by the local government or fisherman’s association in northern and southern Taiwan. Prayer rooms in the harbor region generally have indoor spaces that can hold a small crowd and be used for religious or social activities.

Lastly, we found that information penetration of the central 1955 Hotline was highly effective among Indonesian workers. The 1955 Hotline was established in 2009 by the Ministry of Labor specifically for blue-collar migrant workers who sought help to resolve issues of employment, labor rights, and interpreting services, as well as general counseling for life in Taiwan. Migrants dial “1955” on any telecommunication device and receive the service free of charge on a 24/7 basis, with language choices in Chinese, English, Thai, Indonesian, Tagalog, and Vietnamese [42]. All participants were aware of this service, and a couple of workers used it personally.

### 4.3. Impacts of the COVID-19 Pandemic

The COVID-19 pandemic has unmasked issues that share salient themes with those faced by the broader global migrant populations [43]. The major components of such issues include income insecurity, isolation in the dormitory, and fear of cluster infection. Homesickness and isolation were prominent stressors that exacerbated psychosocial stress during the pandemic. Our study was conducted during the aftermath of a controversial incident where many migrant workers were forbidden from leaving the dormitory, following an episode of cluster infection in an electronics company. The county government announced the order of isolation to be in effect from 4–28 June 2021, targeting only the migrant worker population. Under the order, workers could only travel between the workplace and the dormitory using transport provided by their employers. As expressed by participants in this study, the isolation in the crowded dormitory rendered the healthy workers fearful of cluster infection and experiencing increased psychosocial stress under the deprivation of freedom and social support. As a result, the lockdown impeded workers’ involvement in religious activities by bereaving them of access to the mosque or public prayer rooms, which crippled their stress-coping strategies. This is particularly concerning since religious coping was found to be protective against the risk of depressive illness among the Muslim cohort [40]. Even though the central government made a late announcement to ascertain the lack of legal basis, the local order had encouraged many companies to adopt similar control measures. A comparable incidence was reported in Singapore with an eight-month-long isolation period [44]. 

### 4.4. Study Limitations

We used the snowball and convenience sampling methods to recruit participants through migrant workers’ social networks and at gathering locations, where participants might have unmeasured clustering characteristics [45]. Therefore, the findings cannot be generalized to populations in different regions or other nationalities in the manufacturing industry due to differences in regional policies, climate, and culture of the source countries. Additionally, participants might be more resourceful with better social networks and support than marginalized subgroups who did not participate in the study. The former might have more positive adaptation experiences and successful coping strategies. Secondly, due to the relatively small sample size, we were not able to evaluate whether we had achieved data saturation for the interview outcomes. Nevertheless, these limitations do not undermine the contribution of the study in providing information that can facilitate positive adaptation for migrants with precarious work conditions. Most identified resources and coping strategies are common and can be accessed by all migrant workers. Variability in adaptation strongly depends on the individual’s attitude towards the utilization of resources.

## 5. Conclusions

This study has provided insights into the acculturation experiences of the under-researched Indonesian manufacturing migrant workers in Taiwan. We identified common acculturative and work-related stressors. And we found that workers were able to adopt coping strategies by capitalizing on resources at the individual, institutional, and governmental policy levels to actively solve problems, increase emotional support, and fortify self-appraisals. These findings have important implications. First, they can be used to further illustrate and assess the pathways of how acculturation influences health outcomes (e.g., injury rate) in future research. Secondly, the current policies that help alleviate acculturative stress should be constantly promoted to ensure universal accessibility. Thirdly, the COVID-19 pandemic has disclosed disparities that exacerbate psychosocial stress, such as limited autonomy, economic distress, poor living environment, and discrimination. Supporting measures should be prioritized for these stressors because they can hardly be coped with at the individual level and require structural changes. Finally, the identified coping strategies and resources could inform policy development to assist with positive adaptation and promote the well-being of the migrant worker population. 

## Figures and Tables

**Table 1:** Perceived acculturation outcomes.

Positive Perceptions	Acculturative Stress
Good workplace relationships Good and complete facilities Flexible food options Comfortable dormitory Access to the prayer room and mosque Access to healthcare	Disappointment at the factory scale after arrival Unharmonious relationships with supervisors Disparity in skills vs. salary Food containing pork Weather/extreme temperature Crowded dormitory (e.g., cannot rest) Language barriers Unfamiliarity with Taiwanese culture Curfew/lock-down during COVID-19 Homesickness (e.g., worried about family) Financial stress (e.g., high living costs)

**Table 2:** Acculturative stressors and their coping strategies.

Acculturative Stressor	Level of Available Resources for Coping		
	Individual *	Institutional	Governmental Policy
Language barriers	Being humorous (A); Asking manpower agency for interpreting service (P)	Hiring manpower agency for interpreting service	
Limited food options	Cooking in the dormitory (P)	Providing Indonesian food or food containing no pork	
Poor health conditions/ injury/physical abuse	Taking a leave to rest (P); Seeking healthcare services (P) Calling 1955 Hotline for physical abuse (P)	Implementing the leave management system	Mandatory enrollment in the National Health Insurance (NHI) program
Others	Engaging in religious activities (E); Attending social groups (E); Seeking family’s or friends’ support (E); Developing positive views (A)	Organizing activities	Building public prayer rooms; Running 1955 Hotline on a 24/7 basis

* (P) = problem-solving coping, (E) = emotion-regulating coping, and (A) = appraisal-oriented coping.

## References

[1] ILO (2021). ILO Global Estimates on International Migrant Workers—Results and Methodology.

[2] Taiwan Ministry of Labor Labor Statistics Search Website (Chinese). https://statfy.mol.gov.tw/.

[3] Hargreaves S., Rustage K., Nellums L.B., McAlpine A., Pocock N., Devakumar D., Aldridge R.W., Abubakar I., Kristensen K.L., Himmels J.W. (2019). Occupational health outcomes among international migrant workers: A systematic review and meta-analysis. Lancet Glob. Health.

[4] Moyce S.C., Schenker M. (2018). Migrant Workers and Their Occupational Health and Safety. Annu. Rev. Publ. Health.

[5] Flynn M.A. (2014). Safety & the Diverse Workforce: Lessons From NIOSH’s Work With Latino Immigrants. Prof. Saf..

[6] Schenker M.B. (2010). A Global Perspective of Migration and Occupational Health. Am. J. Ind. Med..

[7] Lin Y.-H., Liao H.-C., Cheng S.-F., Lee L.-H. (2011). Occupational fatality and injury risks for overseas blue-collar workers in Taiwan. J. Chin. Inst. Ind. Eng..

[8] Nakata A., Ikeda T., Takahashi M., Haratani T., Hojou M., Fujioka Y., Swanson N.G., Araki S. (2006). Impact of psychosocial job stress on non-fatal occupational injuries in small and medium-sized manufacturing enterprises. Am. J. Ind. Med..

[9] Hilton M.F., Whiteford H.A. (2010). Associations between psychological distress, workplace accidents, workplace failures and workplace successes. Int. Arch. Occup. Environ. Health.

[10] Sam D.L., Berry J.W. (2006). The Cambridge Handbook of Acculturation Psychology.

[11] Daly A., Carey R.N., Darcey E., Chih H., LaMontagne A.D., Milner A., Reid A. (2018). Workplace psychosocial stressors experienced by migrant workers in Australia: A cross-sectional study. PLoS ONE.

[12] Lee H., Ahn H., Miller A., Park C.G., Kim S.J. (2012). Acculturative Stress, Work-related Psychosocial Factors and Depression in Korean-Chinese Migrant Workers in Korea. J. Occup. Health.

[13] Liem A., Renzaho A.M.N., Hannam K., Lam A.I.F., Hall B.J. (2021). Acculturative stress and coping among migrant workers: A global mixed-methods systematic review. Appl. Psychol. Health Well Being.

[14] Lazarus R.S. (1999). Stress and Emotion: A New Synthesis.

[15] Lazarus R.S., Lazarus R.S. (1963). Personality and Adjustment.

[16] Berry J.W. (1997). Immigration, Acculturation, and Adaptation. Appl. Psychol. Int. Rev..

[17] Berry J.W., Berry J.W., Poortinga Y.H., Segall M.H., Dasen P.R. (2002). Cross-Cultural Psychology: Research and Applications.

[18] Hajro A., Stahl G.K., Clegg C.C., Lazarova M.B. (2019). Acculturation, coping, and integration success of international skilled migrants: An integrative review and multilevel framework. Hum. Resour. Manag. J..

[19] Cheng C.W., Wu T.C. (2013). An investigation and analysis of major accidents involving foreign workers in Taiwan’s manufacture and construction industries. Safety Sci..

[20] Wu T.N., Liou S.H., Hsu C.C., Chao S.L., Liou S.F., Ko K.N., Yeh W.Y., Chang P.Y. (1997). Epidemiologic study of occupational injuries among foreign and native workers in Taiwan. Am. J. Ind. Med..

[21] Adu P. (2019). A Step-by-Step Guide to Qualitative Data Coding.

[22] Taiwan Ministry of Justice Laws & Regulations Database of Taiwan. https://law.moj.gov.tw/Eng/index.aspx.

[23] Dembe A.E., Erickson J.B., Delbos R.G., Banks S.M. (2005). The impact of overtime and long work hours on occupational injuries and illnesses: New evidence from the United States. Occup. Environ. Med..

[24] Gany F., Dobslaw R., Ramirez J., Tonda J., Lobach I., Leng J. (2011). Mexican Urban Occupational Health in the US: A Population at Risk. J. Commun. Health.

[25] Choi J.B., Thomas M. (2009). Predictive factors of acculturation attitudes and social support among Asian immigrants in the USA. Int J. Soc. Welf.

[26] Swaen G.M.H., van Amelsvoort L.P.G.M., Bultmann U., Slangen J.J.M., Kant I.J. (2004). Psychosocial work characteristics as risk factors for being injured in an occupational accident. J. Occup. Environ. Med..

[27] Shepherd R., Lorente L., Vignoli M., Nielsen K., Peiro J.M. (2021). Challenges influencing the safety of migrant workers in the construction industry: A qualitative study in Italy, Spain, and the UK. Safety. Sci..

[28] Sanou D., O’Reilly E., Ngnie-Teta I., Batal M., Mondain N., Andrew C., Newbold B.K., Bourgeault I.L. (2014). Acculturation and nutritional health of immigrants in Canada: A scoping review. J. Immigr. Minor. Health.

[29] Santoso D.S. (2009). The construction site as a multicultural workplace: A perspective of minority migrant workers in Brunei. Constr. Manag. Econ..

[30] Caplan S. (2007). Latinos, acculturation, and acculturative stress: A dimensional concept analysis. Policy Polit Nurs. Pract..

[31] Magana C.G., Hovey J.D. (2003). Psychosocial stressors associated with Mexican migrant farmworkers in the midwest United States. J. Immigr. Health.

[32] Weishaar H.B. (2010). “You have to be flexible”-Coping among polish migrant workers in Scotland. Health Place.

[33] Fischer P., Ai A.L., Aydin N., Frey D., Haslam S.A. (2010). The Relationship Between Religious Identity and Preferred Coping Strategies: An Examination of the Relative Importance of Interpersonal and Intrapersonal Coping in Muslim and Christian Faiths. Rev. Gen. Psychol.

[34] Chang H.C., Wang M.C., Liao H.C., Cheng S.F., Wang Y.H. (2016). Hazard Prevention Regarding Occupational Accidents Involving Blue-Collar Foreign Workers: A Perspective of Taiwanese Manpower Agencies. Int. J. Environ. Res. Public Health.

[35] Guerin P.J., Vold L., Aavitsland P. (2005). Communicable disease control in a migrant seasonal workers population: A case study in Norway. Euro. Surveill.

[36] Pithara C., Zembylas M., Theodorou M. (2012). Access and effective use of healthcare services by temporary migrants in Cyprus. Int. J. Migr. Health Soc. Care.

[37] Tsai Y.C., Wu N.C., Su H.C., Hsu C.C., Guo H.R., Chen K.T. (2020). Differences in injury and trauma management between migrant workers and citizens. Medicine.

[38] Wang M.S., Chan T.C. (2017). The intersections of the care regime and the migrant care worker policy: The example of Taiwan. Asia Pac. J. Soc. Work.

[39] Subchi I., Jahar A.S., Rahiem M.D., Ni’am Sholeh A. (2022). Negotiating Religiosity in a Secular Society: A Study of Indonesian Muslim Female Migrant Workers in Hong Kong. J. Popul. Soc. Stud..

[40] Thomas J., Barbato M. (2020). Positive Religious Coping and Mental Health among Christians and Muslims in Response to the COVID-19 Pandemic. Religions.

[41] Taiwan Tourism Bureau Muslim-friendly Environment. https://eng.taiwan.net.tw/m1.aspx?sNo=0020308.

[42] Taiwan Ministry of Labor For Foreign Workers to Work in Taiwan. https://www.wda.gov.tw/.

[43] Jones K., Mudaliar S., Piper N. (2021). Locked Down and in Limbo: The Global Impact of COVID-19 on Migrant Worker Rights and Recruitment.

[44] Hennebry J., Hari K.C. (2020). Quarantined! Xenophobia and Migrant Workers during the COVID-19 Pandemic.

[45] Johnston L.G., Sabin K. (2010). Sampling Hard-to-Reach Populations with Respondent Driven Sampling. Methodol. Innov. Online.

